# The clinical utility and cost impact of cystatin C measurement in the diagnosis and management of chronic kidney disease: A primary care cohort study

**DOI:** 10.1371/journal.pmed.1002400

**Published:** 2017-10-10

**Authors:** Adam Shardlow, Natasha J. McIntyre, Simon D. S. Fraser, Paul Roderick, James Raftery, Richard J. Fluck, Christopher W. McIntyre, Maarten W. Taal

**Affiliations:** 1 Renal Unit, Royal Derby Hospital, Derby, United Kingdom; 2 Centre for Kidney Research and Innovation, Division of Medical Sciences and Graduate Entry Medicine, School of Medicine, University of Nottingham, Royal Derby Hospital, Derby, United Kingdom; 3 Academic Unit of Primary Care and Population Sciences, Faculty of Medicine, University of Southampton, Southampton, United Kingdom; 4 Division of Nephrology, Schulich School of Medicine and Dentistry, University of Western Ontario, London, Ontario, Canada; Istituto Di Ricerche Farmacologiche Mario Negri, ITALY

## Abstract

**Background:**

To reduce over-diagnosis of chronic kidney disease (CKD) resulting from the inaccuracy of creatinine-based estimates of glomerular filtration rate (GFR), UK and international guidelines recommend that cystatin-C-based estimates of GFR be used to confirm or exclude the diagnosis in people with GFR 45–59 ml/min/1.73 m^2^ and no albuminuria (CKD G3aA1). Whilst there is good evidence for cystatin C being a marker of GFR and risk in people with CKD, its use to define CKD in this manner has not been evaluated in primary care, the setting in which most people with GFR in this range are managed.

**Methods and findings:**

A total of 1,741 people with CKD G3a or G3b defined by 2 estimated GFR (eGFR) values more than 90 days apart were recruited to the Renal Risk in Derby study between June 2008 and March 2010. Using Chronic Kidney Disease Epidemiology Collaboration (CKD-EPI) equations, we compared GFR estimated from creatinine (eGFR_creat_), cystatin C (eGFR_cys_), and both (eGFR_creat-cys_) at baseline and over 5 years of follow-up. We analysed the proportion of participants with CKD G3aA1 reclassified to ‘no CKD’ or more advanced CKD with the latter two equations. We further assessed the impact of using cystatin-C-based eGFR in risk prediction equations for CKD progression and all-cause mortality and investigated non-GFR determinants of eGFR_cys_. Finally, we estimated the cost implications of implementing National Institute for Health and Care Excellence (NICE) guidance to use eGFR_cys_ to confirm the diagnosis in people classified as CKD G3aA1 by eGFR_creat_. Mean eGFR_cys_ was significantly lower than mean eGFR_creat_ (45.1 ml/min/1.73 m^2^, 95% CI 44.4 to 45.9, versus 53.6 ml/min/1.73 m^2^, 95% CI 53.0 to 54.1, *P <* 0.001). eGFR_cys_ reclassified 7.7% (50 of 653) of those with CKD G3aA1 by eGFR_creat_ to eGFR ≥ 60 ml/min/1.73 m^2^. However, a much greater proportion (59.0%, 385 of 653) were classified to an eGFR category indicating more severe CKD. A similar pattern was seen using eGFR_creat-cys_, but lower proportions were reclassified. Change in eGFR_creat_ and eGFR_cys_ over 5 years were weakly correlated (*r* = 0.33, *P <* 0.001), but eGFR_cys_ identified more people as having CKD progression (18.2% versus 10.5%). Multivariable analysis using eGFR_creat_ as an independent variable identified age, smoking status, body mass index, haemoglobin, serum uric acid, serum albumin, albuminuria, and C reactive protein as non-GFR determinants of eGFR_cys_. Use of eGFR_cys_ or eGFR_creat-cys_ did not improve discrimination in risk prediction models for CKD progression and all-cause mortality compared to similar models with eGFR_creat_. Application of the NICE guidance, which assumed cost savings, to participants with CKD G3aA1 increased the cost of monitoring by £23 per patient, which if extrapolated to be applied throughout England would increase the cost of testing and monitoring CKD by approximately £31 million per year. Limitations of this study include the lack of a measured GFR and the potential lack of ethnic diversity in the study cohort.

**Conclusions:**

Implementation of current guidelines on eGFR_cys_ testing in our study population of older people in primary care resulted in only a small reduction in diagnosed CKD but classified a greater proportion as having more advanced CKD than eGFR_creat_. Use of eGFR_cys_ did not improve risk prediction in this population and was associated with increased cost. Our data therefore do not support implementation of these recommendations in primary care. Further studies are warranted to define the most appropriate clinical application of eGFR_cys_ and eGFR_creat-cys_.

## Abstract 2

Adam Shardlow and colleagues investigate whether using cystatin C in addition to serum creatinine can improve diagnosis and risk prediction for progression of CKD among patients in primary care, and estimate the costs of additional testing and monitoring.

## Author summary

**Why was this study done?:**

- Estimation of kidney function (glomerular filtration rate) from serum creatinine concentration may be inaccurate in some people due to the impact of muscle mass, diet, and drugs on creatinine concentration.
- To reduce over-diagnosis of chronic kidney disease, international and UK guidelines recommend that the diagnosis of chronic kidney disease should be confirmed with an estimate of kidney function based on a different marker of glomerular filtration, cystatin C, in those people with only a mild reduction in glomerular filtration rate and no albuminuria.
- The clinical utility of this guidance has not been adequately evaluated in primary care, the setting in which most people with chronic kidney disease are cared for.

**What did the researchers do and find?:**

- We estimated glomerular filtration rate from serum creatinine and cystatin C in a cohort of 1,741 mainly older people diagnosed with chronic kidney disease in primary care.
- The use of cystatin C to confirm a diagnosis of chronic kidney disease resulted in reclassification of a small proportion (7.7%) of people as not having chronic kidney disease, but a much greater proportion were reclassified as having more advanced disease (59%).
- In this cohort, the use of cystatin C did not result in improved risk prediction for all-cause mortality or progression of chronic kidney disease.
- We estimate that the use of cystatin C as recommended in current guidelines would result in increased healthcare costs of £23 per person in the first year of implementation.

**What do these findings mean?:**

- Our data do not support the use of cystatin C to confirm a diagnosis of chronic kidney disease in primary care.
- Cystatin C may be useful for estimating glomerular filtration rate in other settings where creatinine is known to be unreliable, for example in people with extremes of body habitus.

## Introduction

The use of serum creatinine concentration to estimate glomerular filtration rate (GFR) has become widely adopted as the principal test for the diagnosis of chronic kidney disease (CKD). However, the dependence of serum creatinine on muscle mass and the tendency of creatinine-based equations to underestimate GFR at values close to the diagnostic threshold of 60 ml/min/1.73 m^2^ has raised concerns about the risk of over-diagnosis in otherwise healthy older populations when relying on this method and has prompted calls to identify more reliable endogenous filtration markers for the estimation of GFR [1]. Concern has also been expressed that the use of GFR estimated from creatinine but not corrected for age may result in under-diagnosis of CKD in younger people [2]. Cystatin C, a protein that normally crosses the glomerular filtration barrier, has been proposed as an alternative endogenous marker. Cystatin C is produced by all nucleated cells, and is therefore less influenced by muscle mass than creatinine [1,3,4]. Though estimation of GFR from cystatin C alone was found to be no more accurate than creatinine, estimated GFR (eGFR) derived from a combined creatinine and cystatin C equation was more accurate and showed greater precision than eGFR derived from creatinine or cystatin C alone [5].

National Institute for Health and Care Excellence (NICE) and Kidney Disease Improving Global Outcomes (KDIGO) guidance for the diagnosis of CKD stage 3 have recommended use of cystatin-C-based eGFR to confirm or exclude a diagnosis in those found to have a creatinine-based eGFR between 45 and 59 ml/min/1.73 m^2^ and no albuminuria (CKD G3aA1) [6,7]. However, the clinical impact and cost of implementing this recommendation has not been adequately evaluated in the population in which it will be applied: those with mildly reduced eGFR, managed predominantly in primary care. This is important because this group represents the majority of people defined as having CKD. Population-based studies have reported that 3.6% of adults in the US [8] and 3.2% of adults in the UK are in CKD stage G3aA1 [9]. Additionally, whilst cystatin C is not dependent on muscle mass, it has been reported to have other non-GFR determinants including sex, inflammation, obesity, diabetes, smoking, and thyroid dysfunction that may adversely affect GFR estimation in some populations [10–14].

Cystatin C has also been shown to improve discrimination in equations to predict adverse outcomes in CKD stage 3 including end-stage kidney disease (ESKD) [15], all-cause mortality [15], and cardiovascular mortality [16]. Potentially, therefore, its use in the diagnosis and continuing evaluation of people with CKD in primary care may improve our ability to detect individuals at high risk of adverse outcomes, to facilitate targeted monitoring and intervention including early referral to a nephrology service [17]. However, as yet there is little published evidence regarding the use of cystatin-C-based estimates of GFR for risk assessment in primary care.

In this analysis, we aimed to assess the impact of use of cystatin-C-based and combined creatinine and cystatin C eGFR compared to standard creatinine-based estimates in a primary care population with baseline CKD stage 3, defined by 2 measures of GFR more than 90 days apart, and to evaluate the non-GFR determinants of cystatin-C-based eGFR. Additionally, we compared creatinine- and cystatin-C-based estimates of GFR over 5 years of follow-up and evaluated the prognostic accuracy of cystatin C in risk prediction. Finally, we evaluated the cost implications of implementing NICE guidance to confirm a diagnosis of CKD G3aA1 based on creatinine eGFR (eGFR_creat_) by checking cystatin C eGFR (eGFR_cys_) and also considered the use of creatinine and cystatin C eGFR (eGFR_creat-cys_) as an alternative strategy.

## Methods

### Ethics

The Renal Risk in Derby (RRID) study was approved by the Nottingham Research Ethics Committee 1, and is included in the National Institute for Health Research Clinical Research Network Portfolio (NIHR Study ID. 6632). All participants provided written informed consent at study baseline, and repeated the consent at the year 5 study visit. The RRID study complies with the Declaration of Helsinki and the principles of good clinical practice.

### Participants

**Fig 1:**
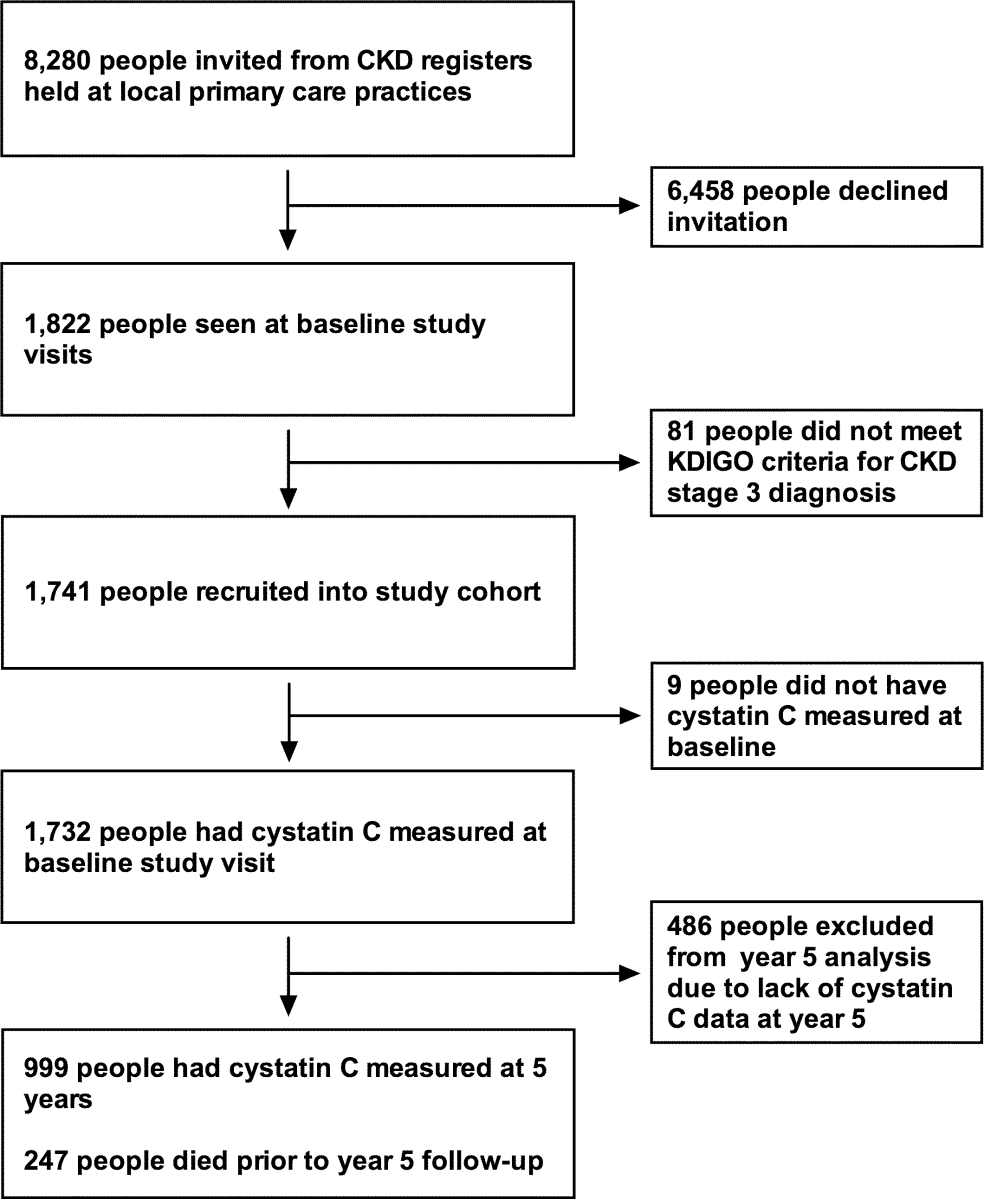
Flow chart for participants invited and recruited into the study, and numbers involved at key time points. CKD, chronic kidney disease; KDIGO, Kidney Disease Improving Global Outcomes.

Detailed methods for the RRID study have been published previously [18]. The study protocol and STROBE and STARD checklists are also available (S1 Protocol; S1 STROBE Checklist; S1 STARD Checklist). In all, 1,741 participants were individually recruited and prospectively studied from 32 Derbyshire primary care practices between June 2008 and March 2010. To start, 8,280 people were invited from practice registers of patients with CKD stage 3. Of these, 1,822 people attended baseline visits. All participants were aged over 18 years. Participants were selected using the 4-variable Modification of Diet in Renal Disease (MDRD) equation modified for use with isotope dilution mass spectrometry–standardised creatinine measurement. Two MDRD eGFR results consistent with CKD stage 3 (30–59 ml/min/1.73 m^2^) more than 90 days apart were required to be eligible. People who were judged to have a life expectancy of less than 1 year, were unable to attend study visits at their primary care surgery, or had previously received a solid organ transplant were excluded from the study. Of the 1,822 people who attended baseline visits, 1,741 were eligible and therefore included in the study cohort (Fig 1).

### Study visits

Study visits were conducted at baseline and repeated at 1 and 5 years. Prior to each visit, participants completed a background questionnaire covering demographic details, medical history, smoking history, and medication history. Participants’ responses to questions were reviewed at the study visit and clarified as required. At each clinical visit, the participant’s height, weight, and waist and hip circumference were measured. Three blood pressure measurements were taken using an oscillometric device (UA-767 Plus 30, A&D Medical) after at least 5 minutes of rest. Readings were repeated until values differed by no more than 10%.

### Laboratory methods

Participants collected 3 consecutive days’ early morning urine samples and stored these in a refrigerator prior to their study visit for subsequent albumin and creatinine analysis. The mean urine albumin-to-creatinine ratio (uACR) from the 3 specimens was used for analysis. Blood samples were taken at each study visit. Participants were asked to abstain from eating meat for 12 hours prior to the study visit to avoid confounding the serum creatinine assay [19]. Blood and urine samples were analysed in a single clinical laboratory at the Royal Derby Hospital for standard haematological and biochemical variables. Creatinine was measured using a compensated Jaffe method, standardised against an isotope dilution mass spectrometry method, with an inter-assay coefficient of variance of 2.3% at 96 mmol/l (Roche P-analyser, Roche Diagnostics). Cystatin C was measured from serum samples taken at baseline, year 1, and year 5 study visits, stored at −80°C. Measurement was undertaken at the biochemistry laboratory at the John Radcliffe Hospital, Oxford, UK, using a particle-enhanced turbidimetric immunoassay assay (Abbott c16000 Analyser, Abbott Diagnostics) calibrated against the international reference material ERM-DA471/IFCC.63. The assay used has a coefficient of variation of 1.5% at 0.89 mg/l and 1.1% at 4.06 mg/l.

### Estimating equations

This analysis compared GFR estimated using the creatinine-based, cystatin-C-based, and combined equations developed by the Chronic Kidney Disease Epidemiology Collaboration (CKD-EPI), designated eGFR_creat_, eGFR_cys_, and eGFR_creat-cys_, respectively [5,20].

### Outcome definitions

We used KDIGO definitions to classify participants’ CKD stage according to eGFR_creat_, eGFR_cys_, and eGFR_creat-cys_. The study prespecified endpoint for CKD progression was the development of ESKD or doubling of serum creatinine. However, this endpoint was observed in only 4 participants (0.2%) after 5 years [21], and we therefore used the KDIGO definition of CKD progression, which is a 25% or more loss of GFR coupled with a worsening of eGFR category or a worsening of albuminuria category [6]. Date and cause of death as stated on death certificates was obtained from the Office for National Statistics via the Health and Social Care Information Centre.

### Statistical analysis

Analysis was conducted according to a prospective analysis plan (see S1 Protocol and S1 Text). Baseline variables were compared according to quartiles of cystatin C, using ANOVA, Kruskal–Wallis, or chi-squared tests as appropriate. Participants were classified according to KDIGO eGFR category initially using eGFR_creat_. Reclassification was undertaken using both eGFR_cys_ and eGFR_creat-cys_. Bland–Altman plots were produced to measure the difference between eGFR_creat_ and both eGFR_cys_ and eGFR_creat-cys_ across the range of eGFR values. Multivariable linear regression models were constructed using eGFR_cys_ as the dependent variable and eGFR_creat_ as well as clinical variables previously reported as non-GFR determinants of cystatin C as covariates. Non-normally distributed variables (uACR, high-sensitivity C-reactive protein [hsCRP]) were logarithmically transformed prior to multivariable analysis.

We have previously reported multivariable models predicting risk of CKD progression (using the KDIGO definition) and all-cause mortality developed in this cohort [21]. Comparison of these models was undertaken using eGFR_cys_ and eGFR_creat-cys_ in place of eGFR_creat_. Binomial logistic regression models were compared using area under the receiver operating characteristic curve (AUROC) based upon predicted probability of progression.

### Cost impact analysis

We used the findings of this study to estimate the cost consequences of implementing cystatin C testing and subsequent monitoring for 12 months as recommended in NICE CKD guidelines for patients with CKD G3aA1 [7]. We assumed that the re-categorising of patients led to the following changes in monitoring by reclassified group: (i) for those classified CKD G3a (no CKD, with diabetes), monitoring continued unchanged, with general practitioner (GP) annual follow-up (eGFR and uACR testing), as recommended by NICE; (ii) for those reclassified as no CKD and without diabetes, the eGFR and uACR tests were dropped from routine monitoring; (iii) for those reclassified as G3b, additional monitoring was added, with eGFR and uACR testing every 6 months via an additional practice nurse consultation; (iv) for those reclassified as G4 or G5, each had a new nephrology outpatient consultation with detailed blood testing and ultrasound, followed by biannual eGFR and uACR testing. The relevant unit costs are shown in Table 1, using costs published by NICE updated to 2015 prices [7].

**Table 1:** Unit costs.

Unit costs derived from NICE^1^, updated to 2015	Amount in British pounds
GP annual (simple) consultation for eGFR and uACR	37.50
Practice nurse consultation with phlebotomy	13.23
eGFR and uACR test	6.19
Nephrology first outpatient consultation including ultrasound scan	292.77

^1^Chronic kidney disease guideline appendices A–R [22].

eGFR, estimated glomerular filtration rate; GP, general practitioner; NICE, National Institute for Health and Care Excellence; uACR, urine albumin-to-creatinine ratio.

## Results

### Baseline data

Cystatin C was measured from stored samples in 1,732 participants at baseline. Baseline values for key variables for this cohort are given in Table 2. Mean ± standard deviation values for eGFR_creat_, eGFR_cys_, and eGFR_creat-cys_ were 53.6 ± 11.8, 45.1 ± 16.0, and 48.3 ± 12.9 ml/min/1.73 m^2^, respectively (*P* < 0.001 for eGFR_cys_ and eGFR_creat-cys_ versus eGFR_creat_). Higher cystatin C was associated with male sex, higher prevalence of previous cardiovascular disease and diabetes mellitus, greater body mass index (BMI), greater waist-to-hip ratio, higher systolic blood pressure, and lower diastolic blood pressure. Haemoglobin, total cholesterol, and serum bicarbonate concentration were lower, and serum uric acid and uACR were higher, in quartiles with higher cystatin C (Table 2).

**Table 2:** Baseline variables by quartile of baseline cystatin C.

Variable	All participants (*n =* 1,732)	Participants by cystatin C quartile				*P* value for trend
		Quartile 1 (*n =* 444)	Quartile 2 (*n =* 432)	Quartile 3 (*n =* 431)	Quartile 4 (*n =* 425)	
eGFR_creat_ (ml/min/1.73 m^2^)	53.6 ± 11.8	63.6 ± 9.7	57.4 ± 7.4	51.1 ± 8.1	41.7 ± 8.9	<0.001
eGFR_cys_ (ml/min/1.73 m^2^)	45.1 ± 16.0	65.5 ± 14.4	47.7 ± 3.8	38.6 ± 3.0	27.9 ± 4.9	<0.001
eGFR_creat-cys_ (ml/min/1.73 m^2^)	48.3 ± 12.9	64.0 ± 9.2	51.7 ± 4.3	43.7 ± 4.3	33.2 ± 5.7	<0.001
Age (years)	72.9 + 9.0	68.6 ± 9.0	72.2 ± 8.3	74.4 ± 8.1	76.4 ± 8.8	0.19
Female sex	1,047 (60.4%)	350 (78.8%)	270 (62.5%)	230 (53.4%)	197 (46.4%)	<0.001
Diabetes	292 (16.9%)	43 (9.7%)	60 (13.9%)	92 (21.3%)	97 (22.8%)	<0.001
Current smoker	81 (4.7%)	17 (3.8%)	18 (4.2%)	18 (4.2%)	28 (6.6%)	0.20
Previous CVD	385 (22.2%)	67 (15.1%)	84 (19.4%)	111 (25.8%)	123 (28.9%)	<0.001
Thyroid disorder	217 (12.5%)	61 (13.7%)	58 (13.4%)	44 (10.2%)	54 (12.7%)	0.39
Haemoglobin (g/l)	132 ± 14	135 ± 13	134 ± 13	133 ± 14	128 ± 16	<0.001
Corrected calcium (mmol/l)	2.38 ± 0.10	2.38 ± 0.10	2.38 ± 0.09	2.38 ± 0.10	2.37 ± 0.10	0.37
Phosphate (mmol/l)	1.11 ± 0.18	1.11 ± 0.18	1.10 ± 0.19	1.09 ± 0.16	1.12 ± 0.18	0.07
Albumin (g/l)	40.7 ± 3.2	41.3 ± 3.0	41.0 ± 3.0	40.4 ± 3.1	40.0 ± 3.5	0.28
Bicarbonate (mmol/l)	25.5 ± 2.7	26.1 ± 2.4	25.7 ± 2.5	25.4 ± 2.7	24.9 ± 3.0	<0.001
Total cholesterol (mmol/l)	4.8 ± 1.2	5.1 ± 1.1	4.8 ± 1.1	4.7 ± 1.2	4.5 ± 1.2	0.04
Uric acid (μmol/l)	384 ± 91	334 ± 75	364 ± 76	398 ± 78	443 ± 96	<0.001
BMI (kg/m^2^)	29.0 ± 5.1	28.4 ± 4.9	28.7 ± 4.7	29.3 ± 4.8	29.7 ± 5.9	0.003
Waist-to-hip ratio	0.91 ± 0.09	0.87 ± 0.08	0.90 ± 0.09	0.92 ± 0.08	0.94 ± 0.09	0.001
SBP (mm Hg)	134 ± 18	133 ± 18	134 ± 17	135 ± 18	134 ± 21	0.001
DBP (mm Hg)	73 ± 11	76 ± 11	73 ± 10	73 ± 11	70 ± 11	0.008
uACR (mg/mmol)	0.33 (0.00–1.50)	0.13 (0.00–0.58)	0.16 (0.00–0.97)	0.50 (0.00–2.07)	1.17 (0.15–4.20)	<0.001
hsCRP (mg/l)	2.2 (1.1–4.6)	1.7 (0.8–3.4)	2.0 (1.1–3.6)	2.5 (1.3–5.5)	3.3 (1.7–6.2)	<0.001

Data shown are mean ± standard deviation, number (percent), or median (lower quartile–upper quartile).

BMI, body mass index; creat, creatinine; cys, cystatin C; creat-cys, creatinine and cystatin C; CVD, cardiovascular disease; DBP, diastolic blood pressure; eGFR, estimated glomerular filtration rate; hsCRP, high-sensitivity C-reactive protein; SBP, systolic blood pressure; uACR, urine albumin-to-creatinine ratio.

**Fig 2:**
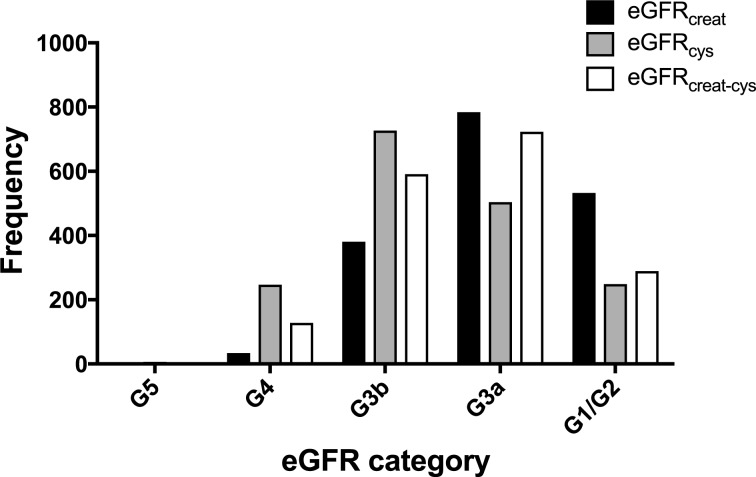
Histogram showing frequency of people in each eGFR category at baseline using different estimating equations. creat, creatinine; cys, cystatin C; creat-cys, creatinine and cystatin C; eGFR, estimated glomerular filtration rate; GFR, glomerular filtration rate.

A comparison of the frequency of people in each eGFR category using the different equations is shown in Fig 2. Fewer participants had a baseline eGFR ≥ 60 ml/min/1.73 m^2^ using either eGFR_cys_ or eGFR_creat-cys_ compared to eGFR_creat_. Similarly, both eGFR_cys_ and eGFR_creat-cys_ classified more participants as having CKD G3b/G4 disease compared to eGFR_creat_.

The use of eGFR_cys_ in the 653 people with eGFR_creat_ CKD G3aA1 at baseline reclassified 50 (7.7%) to eGFR ≥ 60 ml/min/1.73 m^2^ (i.e., no CKD), 356 (54.5%) to G3b, and 29 (4.5%) to G4 or G5. Similarly, using eGFR_creat-cys_ reclassified 36 (5.5%) to no CKD, 239 (36.6%) to G3b, and 2 (0.3%) to G4 or G5 (Table 3). Application of eGFR_cys_ to the whole study population reclassified 57 of 784 (7.3%) with eGFR_creat_ CKD G3a to eGFR ≥ 60 ml/min/1.73 m^2^ and 488 (62.2%) to CKD G3b or worse (Table 4). Similarly, in the whole study population, eGFR_creat-cys_ reclassified 4.7% of participants (37 of 784) with eGFR_creat_ CKD G3a to eGFR≥> 60 ml/min/1.73 m^2^ and 311 (39.7%) to CKD G3b or G4 (Table 5).

**Table 3:** Reclassification in 653 participants classified as CKD G3aA1 by eGFRcreat at baseline using eGFRcys and eGFRcreat-cys.

Estimating equation	eGFR ≥ 60 ml/min/1.73 m^2^	CKD G3a	CKD G3b	CKD 4	CKD 5
eGFR_cys_	50 (7.7%)	218 (33.4%)	356 (54.5%)	28 (4.3%)	1 (0.2%)
eGFR_creat-cys_	36 (5.5%)	376 (57.6%)	239 (36.6%)	2 (6.3%)	0

Data shown are number (percent).

CKD, chronic kidney disease; creat, creatinine; cys, cystatin C; creat-cys, creatinine and cystatin C; eGFR, estimated glomerular filtration rate.

**Table 4:** Baseline eGFRcreat category and reclassification using eGFRcys in all study participants.

Baseline eGFR_creat_ category	eGFR_cys_ category					Total
	G1/G2	G3a	G3b	G4	G5	
G1/G2	182 (34.1%)	251 (47.1%)	96 (18.0%)	4 (0.8%)	0	533 (30.8%)
G3a	57 (7.3%)	239 (30.5%)	446 (56.9%)	41 (5.2%)	1 (0.1%)	784 (45.2%)
G3b	10 (2.6%)	12 (3.1%)	183 (48.0%)	174 (45.7%)	2 (0.5%)	381 (22.0%)
G4	0	2 (5.9%)	2 (5.9%)	28 (82.4%)	2 (5.9%)	34 (2.0%)

Data shown are number (percent). Cohen’s Kappa for agreement between eGFRcreat and eGFRcreat-cys = 0.13.

creat, creatinine; cys, cystatin C; eGFR, estimated glomerular filtration rate.

**Table 5:** Baseline eGFRcreat category and reclassification using eGFRcreat-cys in all study participants.

Baseline eGFR_creat_ category	eGFR_creat-cys_ category					Total
	G1/G2	G3a	G3b	G4	G5	
G1/G2	249 (46.7%)	274 (51.4%)	10 (1.9%)	0	0	533 (30.8%)
G3a	37 (4.7%)	436 (55.6%)	309 (39.4%)	2 (0.3%)	0	784 (45.2%)
G3b	3 (0.8%)	13 (3.4%)	270 (70.9%)	95 (24.9%)	0	381 (22.0%)
G4	0	0	2 (5.9%)	31 (91.2%)	1 (2.9%)	34 (2.0%)

Data shown are number (percent). Cohen’s Kappa for agreement between eGFRcreat and eGFRcreat-cys = 0.37.

creat, creatinine; creat-cys, creatinine and cystatin C; eGFR, estimated glomerular filtration rate.

### Bland–Altman plots

**Fig 3:**
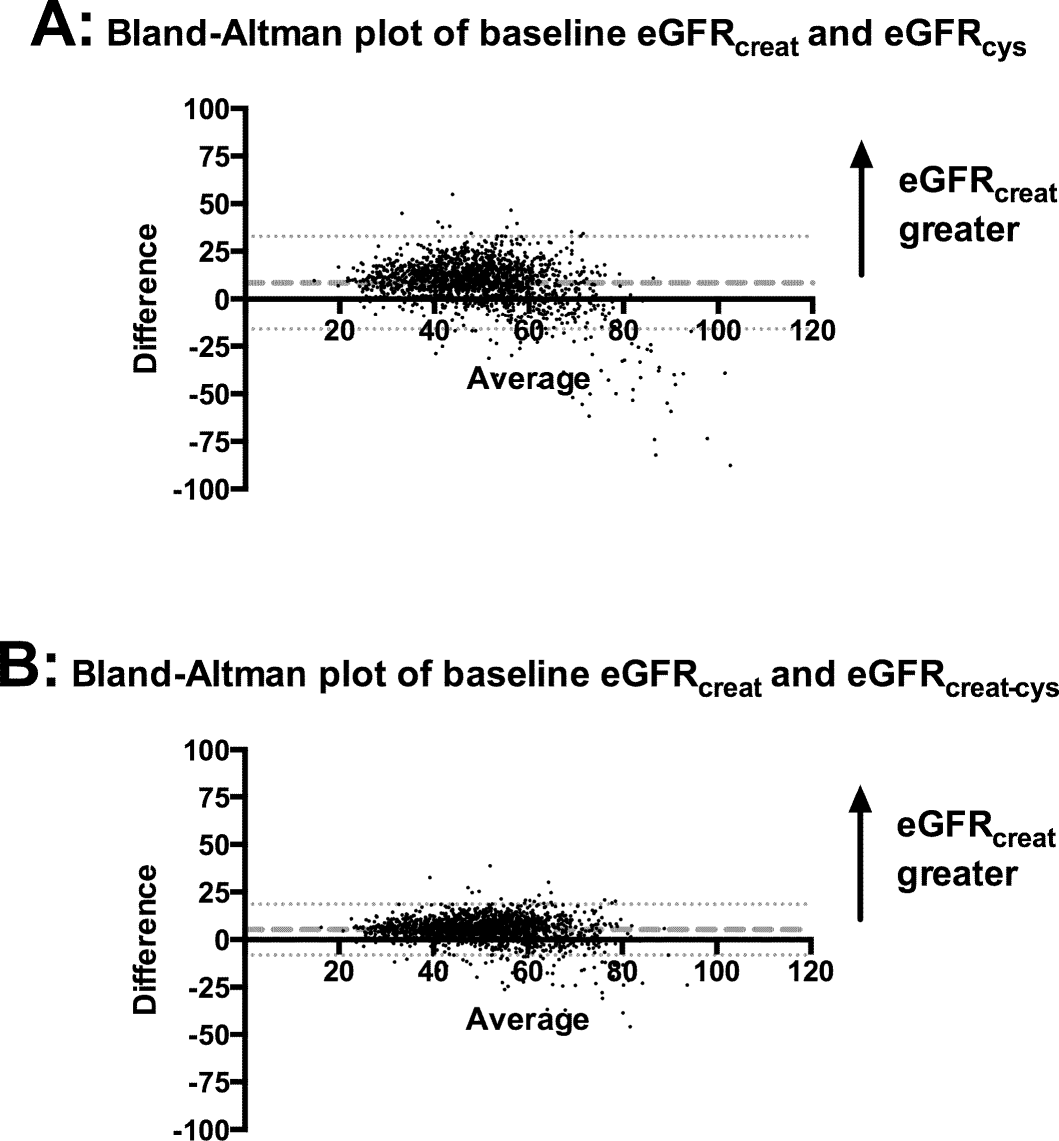
Bland–Altman plots comparing eGFRcreat to eGFRcys and eGFRcreat-cys. (A) eGFRcreat versus eGFRcys; (B) eGFRcreat versus eGFRcreat-cys. Dashed lines show mean difference between the 2 estimates of glomerular filtration rate. Dotted lines show 95% CI for mean difference between the 2 estimates. creat, creatinine; creat-cys, creatinine and cystatin C; cys, cystatin C; eGFR, estimated glomerular filtration rate.

Bland–Altman plots in the whole cohort showed that for the majority of participants, eGFR_creat_ was greater than eGFR_cys_ and eGFR_creat-cys_ (Fig 3). Mean difference was +8.4 ml/min/1.73 m^2^ between eGFR_creat_ and eGFR_cys_ and +5.3 ml/min/1.73 m^2^ between eGFR_creat_ and eGFR_creat-cys_. Both plots showed a small minority of cases, at higher mean eGFR, for which eGFR_cys_ or eGFR_creat-cys_ was greater than eGFR_creat_.

### Non-GFR determinants of eGFR_cys_

Non-GFR determinants of eGFR_cys_ at baseline were assessed using linear regression, with correction for baseline eGFR_creat_. In fully adjusted models, a range of factors remained significant including age, smoking status, and BMI. Other significant determinants included markers of inflammation and non-traditional cardiovascular risk factors (haemoglobin, uric acid, albumin, uACR, and hsCRP) (S1 Table).

### Change in eGFR over 5 years

Nine hundred ninety-nine participants had cystatin C measured at both baseline and year 5 (Fig 1). There was a weak correlation between 5-year change in eGFR_cys_ and 5-year change in eGFR_creat_ (Pearson’s correlation coefficient, *r* = 0.33, *P <* 0.001), and a moderate correlation between 5-year change in eGFR_creat-cys_ and 5-year change in eGFR_creat_ (*r* = 0.76, *P* < 0.001).

Over 5 years, the KDIGO definition for CKD progression based on 25% loss of eGFR and a worsening of eGFR category or albuminuria category was met in 105 of 999 participants (10.5%) using eGFR_creat_, 182 (18.2%) using eGFR_cys_, and 135 (13.5%) using eGFR_creat-cys_.

### Risk prediction

Overall, 306 participants (17.7%) met the KDIGO criteria for CKD progression at 5 years, and 247 (14.2%) died. Replacing baseline eGFR_creat_ with eGFR_cys_ or eGFR_creat-cys_ in previously developed multivariable prediction models for CKD progression [21] did not improve discrimination. The AUROC was comparable for all 3 models (Table 6). Similarly, in multivariable Cox proportional hazards models for all-cause mortality over 5 years, similar hazard ratios were obtained for eGFR with each estimating equation (Table 6).

**Table 6:** Risk prediction models for CKD progression in 999 participants and all-cause mortality in 1,732 participants using different estimating equations for eGFR.

Risk prediction	Estimating equation	Odds ratio or hazard ratio (95% CI)	*P* value	AUROC
**Models for KDIGO CKD progression**	eGFR_creat_	0.984 (0.971–0.998)	0.023	0.722
	eGFR_cys_	0.982 (0.971–0.993)	0.001	0.726
	eGFR_creat-cys_	0.978 (0.965–0.991)	0.001	0.726
**Models for all-cause mortality**	eGFR_creat_	0.973 (0.960–0.986)	<0.001	
	eGFR_cys_	0.975 (0.963–0.987)	<0.001	
	eGFR_creat-cys_	0.967 (0.954–0.981)	<0.001	

All progression models are adjusted for age, sex, urine albumin-to-creatinine ratio, haemoglobin, bicarbonate, and diabetes. All odds ratios given per ml/min/1.73 m2. All survival models are adjusted for age, sex, urine albumin-to-creatinine ratio, haemoglobin, albumin, bicarbonate, diabetes, and previous cardiovascular disease. All hazard ratios are given per ml/min/1.73 m2.

AUROC, area under the receiver operating characteristic curve; CKD, chronic kidney disease; creat, creatinine; cys, cystatin C; creat-cys, creatinine and cystatin C; eGFR, estimated glomerular filtration rate; KDIGO, Kidney Disease Improving Global Outcomes.

### Cost impact

The impacts on National Health Service (NHS) costs for groups reclassified with CKD G3aA1 by eGFR_cys_ or eGFR_creat-cys_ are summarised in Table 7, based on conservative assumptions. The direct cost of adding eGFR_cys_ testing to existing tests would be fairly low based on NICE’s estimated cost of just over £3 per test. However, the total cost impact of providing recommended monitoring and referral would be much greater, at £20 per person (£12,843 for the 653 persons in this study). Thus, the combined total cost impact would be an increase of £23 (£20 + £3) per person. This impact would be less if using the combined eGFR_creat-cys_ equation, with a total cost of £8 per person. This lower cost results from fewer people being reclassified in either direction (Table 7).

**Table 7:** Cost impact of cystatin C testing in the year of introduction, by GFR estimating equation, at 2015 prices (British pounds).

Outcome	Reclassification status	Reference^1^	Change in cost	Unit cost (£)	Using eGFR_cys_		Using eGFR_creat-cys_	
					Number affected	Total cost (£)	Number affected	Total cost (£)
**Change in management**								
Stop monitoring	G1/G2, no DM, no HT	NICE CKD 182	Decrease	7.50	15	−563	11	−413
Diabetes schedule unchanged	G1/G2, DM	NICE DM 28	Nil		6	0	0	0
Exclude eGFR and uACR test from annual review	G1/G2, HT	NICE HT 127	Decrease	6.19	29	−180	22	−136
Unchanged from annual GP assessment of eGFR and uACR	G3a		Nil		218	0	376	0
Biannual assessment of eGFR and uACR^2^	G3b	NICE CKD 182	Increase	13.23	356	4,711	239	3,163
Nephrology, followed by biannual GP assessment of eGFR and uACR^3^	G4, G5	NICE CKD 182	Increase	306.00	29	8,874	2	612
**Total increase**						12,843		3,226
**Increase £/patient (monitoring)**						20		5
**Total increase £/patient**						23		8

^1^References are NICE guidance documents. Numbers in the column are guideline numbers (see https://www.nice.org.uk/guidance).

^2^Unit cost for biannual assessment assumes this involves 1 additional visit to a practice nurse with phlebotomy for eGFR and uACR testing.

^3^Unit cost for nephrology followed by biannual GP visit assumes this involves 1 extra outpatient consultation plus 1 additional visit to a practice nurse with phlebotomy for eGFR and uACR testing.

CKD, chronic kidney disease; creat-cys, creatinine and cystatin C; cys, cystatin C; DM, diabetes mellitus; eGFR, estimated glomerular filtration rate; GFR, glomerular filtration rate; GP, general practitioner; HT, hypertension; NICE, National Institute for Health and Care Excellence; uACR, urine albumin-to-creatinine ratio.

## Discussion

Our results indicate that for the majority with CKD stage 3 (confirmed by 2 eGFR_creat_ values) in primary care, use of eGFR_cys_ or eGFR_creat-cys_ results in lower estimates of GFR than eGFR_creat_. The use of eGFR_cys_ as recommended by NICE to confirm an eGFR_creat_-based diagnosis of CKD G3aA1 resulted in reclassification of 7.7% as not having CKD, but a far greater proportion (59.0%) were reclassified as having more advanced CKD (G3b–G5). Thus, in a primary care setting, the potential benefit of reducing over-diagnosis of CKD with eGFR_cys_ would be eliminated by the unintended consequence of greater reclassification to more advanced CKD requiring more frequent monitoring and increased referrals to secondary care. Additionally, the use of eGFR_cys_ did not improve discrimination in risk prediction models in a primary care population. Overall estimated costs would be increased by £23 per patient with eGFR_cys_ and £8 per patient with eGFR_creat-cys_.

Our results differ in many respects from those of a large meta-analysis that evaluated the clinical impact of using cystatin C versus creatinine to estimate GFR in 11 general population cohorts and 5 CKD cohorts [23]. In the meta-analysis, though no difference was observed in mean eGFR calculated by the different methods, use of eGFR_cys_ resulted in a higher prevalence of eGFR < 60 ml/min/1.73 m^2^ than either eGFR_creat_ or eGFR_creat-cys_ (13.7%, 9.7%, and 10.0%, respectively). On the other hand, use of eGFR_cys_ resulted in reclassification of 35%–47% of participants with CKD G3a to GFR ≥ 60 ml/min/1.73 m^2^, whereas a lower proportion (21%–27%) were reclassified to CKD G3b or worse. Moreover, reclassification to a less severe category was associated with lower risks of all-cause mortality, cardiovascular mortality, and ESKD [23]. One reason for the differences between these observations and ours is important differences in the cohorts studied. The mean age of 60 and 55 years for the general population and CKD cohorts, respectively, was substantially lower than the mean age of our cohort (73 years). This is an important distinction because our cohort is more representative of the majority of people affected by CKD in developed countries with predominantly white populations. A second important difference is that the studies included in the meta-analysis relied on only 1 abnormal eGFR_creat_ for the diagnosis of CKD, whereas we required confirmation with 2 abnormal eGFR_creat_ values at least 90 days apart for study eligibility. In our study, those classified as CKD G3a at baseline therefore had a minimum of 3 abnormal eGFR_creat_ values. It is likely that simply retesting eGFR_creat_ would have reclassified a proportion of those included in the meta-analysis as not having CKD, and this would reduce the impact of reclassification by eGFR_cys_. In a population-based study in England, use of eGFR_cys_ resulted in a higher prevalence of CKD G3–G5 than eGFR_creat_ (7.7% versus 5.2%, respectively) [17]. In this study, similar to the above meta-analysis, 37% of those with CKD G3aA1 defined by eGFR_creat_ were reclassified by eGFR_cys_ as not having CKD, but the proportion reclassified to an eGFR category indicating more severe CKD was not reported. Like the meta-analysis, the participants in this study were much younger than our cohort (median age 50 years), and only a single creatinine measurement was used to define CKD. In addition, the cystatin C assay used was not standardised to international reference material, and the CKD-EPI equation could not be used [17]. In contrast, an analysis of National Health and Nutrition Examination Survey data revealed higher prevalence of reduced GFR by eGFR_cys_ than eGFR_creat_ in both diabetic and non-diabetic participants [24].

In our study, eGFR_cys_ identified a higher proportion of participants as having progressive CKD (18.2%) than eGFR_creat_ (10.5%) or eGFR_creat-cys_ (13.5%). Thus, in addition to the impact of the lower baseline eGFR values seen with eGFR_cys_, higher apparent progression rates would further promote the referral of patients from primary to secondary care. One could argue that increased referral would be appropriate if patients were at increased risk, but the very low rate of progression to ESKD observed in our study population after 5 years (0.2%) [21] implies that use of eGFR_cys_ in this primary care population would tend to increase referrals and frequency of testing of people with low-risk disease who would be unlikely to benefit.

There is ongoing debate concerning the appropriateness of diagnosing CKD in older people with category G3a eGFR and no proteinuria [25]. In this analysis, we applied current guidelines to diagnosis of CKD in our cohort. We have previously described the low rates of CKD progression and relatively high rates of ‘remission’ in this population [21]. Additionally, recent results from the Berlin Initiative Study (BIS) have shown that eGFR in older adults strongly depends upon the estimating equation used [26]. The BIS equations (creatinine only and combined creatinine and cystatin C) were developed in a cohort of people over the age of 70 years. These equations tend to produce lower eGFR values than the corresponding CKD-EPI equations and are more accurate in predicting measured GFR [27]. Comparable results have been shown using the full age spectrum (FAS) equation [28]. Our study focussed on the CKD-EPI equations as these have been incorporated into KDIGO and NICE guidance and are in widespread use clinically.

Several studies have reported that, like all endogenous markers of GFR, serum cystatin C concentration is independently associated with several non-GFR determinants including age, sex, diabetes, markers of obesity, inflammation, and smoking [10–13]. Though we did not have measured GFR data, multivariable analysis corrected for eGFR_creat_ confirmed independent associations of eGFR_cys_ with age, serum albumin, serum uric acid, haemoglobin, BMI, uACR, hsCRP, and current smoking (S1 Table) [29]. These observations are important because several of these non-GFR determinants are also risk factors for cardiovascular disease, and this may in part explain the better performance of eGFR_cys_ as a risk factor for adverse outcomes in CKD cohorts and populations without CKD. Indeed, some have suggested that the ability of cystatin C concentration to predict mortality may have little to do with its association with GFR but instead is largely attributable to the non-GFR determinants of cystatin C [30,31]. Alternatively, other investigators have proposed that in states of inflammation, filtration of cystatin C at the glomerulus is impaired, producing underestimates of GFR [32]. In addition, understanding the non-GFR determinants of cystatin C is important for identifying patient groups in whom eGFR_cys_ will be unreliable. Our data, though limited by lack of measured GFR, confirm previous reports suggesting that eGFR_cys_ is likely to be less accurate for estimating GFR in elderly and obese patients as well as those with albuminuria or evidence of inflammation and in current smokers. However, there may be situations where measurement of eGFR_cys_ may be preferred to eGFR_creat_, for example in the assessment of renal function in younger people with extremes of body habitus and muscle mass.

Reduced GFR is widely recognised as an independent risk factor for multiple adverse outcomes including acute kidney injury, ESKD, cardiovascular mortality, and all-cause mortality [33–35]. Several papers have reported improved discrimination if eGFR_cys_ is used in risk prediction analyses instead of eGFR_creat_, though it is unclear whether this is due to improved GFR estimation or associations with the non-GFR determinants of cystatin C. In our cohort, eGFR_cys_ did not improve discrimination in risk prediction analyses for CKD progression or all-cause mortality, suggesting that widespread use in primary care will not improve risk prediction [15,16].

Our assessment of the cost associated with implementing NICE guidance to use eGFR_cys_ to confirm a diagnosis in those classified as CKD G3aA1 by eGFR_creat_ resulted in an overall increase in cost of £23 per patient because the cost savings resulting from reduced numbers diagnosed with CKD were far outweighed by the increased costs associated with a requirement for increased monitoring and referral in the large proportion reclassified to a more advanced stage of CKD. The total number of patients meeting the inclusion criteria of this study in the adult population of England can be estimated approximately from the Health Survey for England as 1.36 million (prevalence in adults of G3aA1 of 3.2%) [9]. If the increase of £23 per patient due to implementation of the NICE guidance was applied to each of these patients, the total additional cost to the NHS would be approximately £31 million per year. A lower total national cost of about £11 million would apply if instead the combined eGFR_creat-cys_ equation was used. This cost could potentially be justified if the use of eGFR_cys_ were associated with higher-risk patients being successfully treated with more intensive treatment or referral, but we were unable to demonstrate improved risk prediction in this predominantly low-risk study population. In interpreting these national cost extrapolations, it should be remembered that, like most epidemiological studies, the Health Survey for England measured only a single eGFR value and may therefore have overestimated the true prevalence of CKD G3aA1.

### Study strengths and limitations

Important strengths of this study are individual recruitment and clinical assessment at baseline, prospective protocol-driven follow-up, and a requirement for 2 eGFR readings of <60 ml/min/1.73 m^2^ prior to inclusion in the study cohort [36,37]. This last strength is of particular significance because the majority of published studies have adopted the epidemiological study approach of requiring only 1 abnormal eGFR for CKD diagnosis. Moreover, our study population was predominantly elderly, and most participants had only mildly reduced GFR. This is typical of the majority affected by CKD in developed countries [8] and is representative of populations in which NICE and KDIGO anticipated that use of eGFR_cys_ would reduce over-diagnosis of CKD. We were also able to evaluate the short-term cost implication of using the different equations.

We must, however, concede several important limitations of the study. We were limited by the lack of a measured GFR at baseline in order to compare estimating equations to a ‘gold standard’. However, the aim of this study was to assess primarily the clinical impact of introducing eGFR_cys_ in primary care, where few people have a measured GFR, rather than the accuracy of the estimating equations. Few people with CKD have a measured GFR, and our study therefore reflects the situation in clinical practice. The lack of a measured GFR also impacts upon our assessment of non-GFR determinants of eGFR_cys_ due to potential confounding by non-GFR determinants of eGFR_creat_ (used as a correction in the analysis instead of measured GFR). Nevertheless, our results are consistent with previous published studies and strengthen the evidence by showing that non-GFR determinants of cystatin C are an important consideration in the primary care setting. The risk prediction models described in this paper were used to show that the use of eGFR_cys_ did not improve discrimination compared to eGFR_creat_ in this cohort. It was not our intention to develop risk prediction models for general application, and we concede that external validation would be required before this could be recommended. Our study population was predominantly white and elderly (mean age 73 years), and most had only mild reductions in GFR (mean eGFR_creat_ 53.6 ml/min/1.73 m^2^). As discussed above, this is in some respects a strength, but we concede that our results may not be applicable to younger or more ethnically diverse populations or to those in secondary care with more advanced CKD. The number of events of death and CKD progression was also relatively low, and we may therefore have lacked statistical power to detect minor improvement in risk prediction with eGFR_cys_. Our cost impact analysis was limited to the year of introduction of cystatin C testing. Lifetime (or long term) costing would require more complex modelling that is beyond the scope of this paper.

### Conclusions

We have found that in an elderly population in primary care, application of NICE and KDIGO recommendations to use eGFR_cys_ to confirm a diagnosis of CKD in those classified as CKD G3aA1 by eGFR_creat_ results in a greater proportion of individuals being reclassified to an eGFR category indicating more severe CKD than reclassified to an eGFR category indicating no CKD. Additionally, eGFR_cys_ cannot be recommended to improve risk prediction in this population because it did not improve discrimination in risk prediction models for adverse outcomes compared to eGFR_creat_. Our data therefore do not support implementation of these recommendations in primary care. Nevertheless, it is likely that eGFR_cys_ will be helpful in obtaining a more accurate estimate of GFR in people at extremes of muscle mass, in whom eGFR_creat_ is known to be inaccurate, but account should also be taken of the non-GFR determinants of cystatin C. Further studies are warranted to define the most appropriate clinical application of eGFR_cys_ and eGFR_creat-cys_.

## Supporting information

S1 STARD Checklist STARD statement for reporting of diagnostic accuracy studies. (DOCX)

S1 STROBE Checklist STROBE statement for reporting of cohort studies. (DOCX)

S1 Table Univariate and multivariable non-GFR determinants of eGFR_cys_. (DOCX)

S1 Protocol Current study protocol. (DOC)

S1 Text Application submitted for funding from the Dunhill medical trust. (DOC)

## Abbreviations

AUROC: area under the receiver operating characteristic curve
BMI: body mass index
CKD: chronic kidney disease
CKD-EPI: Chronic Kidney Disease Epidemiology Collaboration
creat: creatinine
creat-cys: creatinine and cystatin C
CVD: cardiovascular disease
cys: cystatin C
DBP: diastolic blood pressure
eGFR: estimated glomerular filtration rate
ESKD: end-stage kidney disease
GFR: glomerular filtration rate
GP: general practitioner
hsCRP: high-sensitivity C-reactive protein
KDIGO: Kidney Disease Improving Global Outcomes
NHS: National Health Service
NICE: National Institute for Health and Care Excellence
RRID: Renal Risk in Derby
SBP: systolic blood pressure
uACR: urine albumin-to-creatinine ratio

## References

[1] Levey AS, Fan L, Eckfeldt JH, Inker LA. Cystatin C for glomerular filtration rate estimation: coming of age. Clin Chem. 2014;60(7):916–9. doi: 10.1373/clinchem.2014.225383 24871681 10.1373/clinchem.2014.225383

[2] Delanaye P, Glassock RJ, Pottel H, Rule AD. An age-calibrated definition of chronic kidney disease: rationale and benefits. Clin Biochem Rev. 2016;37(1):17–26. 27057075 PMC4810758

[3] Ferguson TW, Komenda P, Tangri N. Cystatin C as a biomarker for estimating glomerular filtration rate. Curr Opin Nephrol Hypertens. 2015;24(3):295–300. doi: 10.1097/MNH.0000000000000115 26066476 10.1097/MNH.0000000000000115

[4] Stevens LA, Schmid CH, Zhang YL, Coresh J, Manzi J, Landis R, et al Development and validation of GFR-estimating equations using diabetes, transplant and weight. Nephrol Dial Transplant. 2010;25(2):449–57. doi: 10.1093/ndt/gfp510 19793928 10.1093/ndt/gfp510 PMC2910328

[5] Inker LA, Schmid CH, Tighiouart H, Eckfeldt JH, Feldman HI, Greene T, et al Estimating glomerular filtration rate from serum creatinine and cystatin C. N Engl J Med. 2012;367(1):20–9. doi: 10.1056/NEJMoa1114248 22762315 10.1056/NEJMoa1114248 PMC4398023

[6] KDIGO 2012 clinical practice guidelines for the evaluation and management of chronic kidney disease. Kidney Int Suppl (2011). 2013;3(1):1–150. 10.1038/ki.2013.243 23989362

[7] National Institute for Health and Care Excellence. Chronic kidney disease in adults: assessment and management. Clinical guideline [CG182]. London: National Institute for Health and Care Excellence; 2014.

[8] Levey AS, Coresh J. Chronic kidney disease. Lancet. 2012;379(9811):165–80. doi: 10.1016/S0140-6736(11)60178-5 21840587 10.1016/S0140-6736(11)60178-5

[9] Fraser SD, Roderick PJ, Aitken G, Roth M, Mindell JS, Moon G, et al Chronic kidney disease, albuminuria and socioeconomic status in the Health Surveys for England 2009 and 2010. J Public Health (Oxf). 2014;36(4):577–86. doi: 10.1093/pubmed/fdt117 24277777 10.1093/pubmed/fdt117

[10] Stevens LA, Schmid CH, Greene T, Li L, Beck GJ, Joffe MM, et al Factors other than glomerular filtration rate affect serum cystatin C levels. Kidney Int. 2009;75(6):652–60. doi: 10.1038/ki.2008.638 19119287 10.1038/ki.2008.638 PMC4557800

[11] Rule AD, Bailey KR, Lieske JC, Peyser PA, Turner ST. Estimating the glomerular filtration rate from serum creatinine is better than from cystatin C for evaluating risk factors associated with chronic kidney disease. Kidney Int. 2013;83(6):1169–76. doi: 10.1038/ki.2013.7 23423253 10.1038/ki.2013.7 PMC3661736

[12] Mathisen UD, Melsom T, Ingebretsen OC, Jenssen T, Njolstad I, Solbu MD, et al Estimated GFR associates with cardiovascular risk factors independently of measured GFR. J Am Soc Nephrol. 2011;22(5):927–37. doi: 10.1681/ASN.2010050479 21454717 10.1681/ASN.2010050479 PMC3083314

[13] Knight EL, Verhave JC, Spiegelman D, Hillege HL, de Zeeuw D, Curhan GC, et al Factors influencing serum cystatin C levels other than renal function and the impact on renal function measurement. Kidney Int. 2004;65(4):1416–21. doi: 10.1111/j.1523-1755.2004.00517.x 15086483 10.1111/j.1523-1755.2004.00517.x

[14] Liu X, Foster MC, Tighiouart H, Anderson AH, Beck GJ, Contreras G, et al Non-GFR determinants of low-molecular-weight serum protein filtration markers in CKD. Am J Kidney Dis. 2016;68(6):892–900. doi: 10.1053/j.ajkd.2016.07.021 27663042 10.1053/j.ajkd.2016.07.021 PMC5123901

[15] Peralta CA, Shlipak MG, Judd S, Cushman M, McClellan W, Zakai NA, et al Detection of chronic kidney disease with creatinine, cystatin C, and urine albumin-to-creatinine ratio and association with progression to end-stage renal disease and mortality. JAMA. 2011;305(15):1545–52. doi: 10.1001/jama.2011.468 21482744 10.1001/jama.2011.468 PMC3697771

[16] Peralta CA, Katz R, Sarnak MJ, Ix J, Fried LF, De Boer I, et al Cystatin C identifies chronic kidney disease patients at higher risk for complications. J Am Soc Nephrol. 2011;22(1):147–55. doi: 10.1681/ASN.2010050483 21164029 10.1681/ASN.2010050483 PMC3014043

[17] Fraser SD, Aitken G, Taal MW, Mindell JS, Moon G, Day J, et al Exploration of chronic kidney disease prevalence estimates using new measures of kidney function in the health survey for England. PLoS ONE. 2015;10(2):e0118676 doi: 10.1371/journal.pone.0118676 25700182 10.1371/journal.pone.0118676 PMC4336286

[18] McIntyre NJ, Fluck RJ, McIntyre CW, Taal MW. Risk profile in chronic kidney disease stage 3: older versus younger patients. Nephron Clin Pract. 2011;119(4):c269–76. doi: 10.1159/000329109 21921639 10.1159/000329109

[19] Preiss DJ, Godber IM, Lamb EJ, Dalton RN, Gunn IR. The influence of a cooked-meat meal on estimated glomerular filtration rate. Ann Clin Biochem. 2007;44(Pt 1):35–42. doi: 10.1258/000456307779595995 17270090 10.1258/000456307779595995

[20] Levey AS, Stevens LA, Schmid CH, Zhang YL, Castro AF 3rd, Feldman HI, et al A new equation to estimate glomerular filtration rate. Ann Intern Med. 2009;150(9):604–12. 19414839 10.7326/0003-4819-150-9-200905050-00006 PMC2763564

[21] Shardlow A, McIntyre NJ, Fluck RJ, McIntyre CW, Taal MW. Chronic kidney disease in primary care: outcomes after five years in a prospective cohort study. PLoS Med. 2016;13(9):e1002128 doi: 10.1371/journal.pmed.1002128 27648564 10.1371/journal.pmed.1002128 PMC5029805

[22] National Institute for Health and Care Excellence. Chronic kidney disease (partial update): early identification and management of chronic kidney disease in adults in primary and secondary care. Guideline appendices: appendix A–R. London: National Institute for Health and Care Excellence; 2014 Jul [cited 2017 Sep 19]. Available from: https://www.nice.org.uk/guidance/cg182/evidence/appendices-a-r-pdf-191905166 25340245

[23] Shlipak MG, Matsushita K, Arnlov J, Inker LA, Katz R, Polkinghorne KR, et al Cystatin C versus creatinine in determining risk based on kidney function. N Engl J Med. 2013;369(10):932–43. doi: 10.1056/NEJMoa1214234 24004120 10.1056/NEJMoa1214234 PMC3993094

[24] Tsai CW, Grams ME, Inker LA, Coresh J, Selvin E. Cystatin C- and creatinine-based estimated glomerular filtration rate, vascular disease, and mortality in persons with diabetes in the U.S. Diabetes Care. 2014;37(4):1002–8. doi: 10.2337/dc13-1910 24271191 10.2337/dc13-1910 PMC3964484

[25] Glassock R, Denic A, Rule AD. When kidneys get old: an essay on nephro-geriatrics. J Bras Nefrol. 2017;39(1):59–64. doi: 10.5935/0101-2800.20170010 28355403 10.5935/0101-2800.20170010

[26] Ebert N, Jakob O, Gaedeke J, van der Giet M, Kuhlmann MK, Martus P, et al Prevalence of reduced kidney function and albuminuria in older adults: the Berlin Initiative Study. Nephrol Dial Transplant. 2017;32(6):997–1005. doi: 10.1093/ndt/gfw079 27190381 10.1093/ndt/gfw079

[27] Schaeffner ES, Ebert N, Delanaye P, Frei U, Gaedeke J, Jakob O, et al Two novel equations to estimate kidney function in persons aged 70 years or older. Ann Intern Med. 2012;157(7):471–81. doi: 10.7326/0003-4819-157-7-201210020-00003 23027318 10.7326/0003-4819-157-7-201210020-00003

[28] Pottel H, Delanaye P, Schaeffner E, Dubourg L, Eriksen BO, Melsom T, et al Estimating glomerular filtration rate for the full age spectrum from serum creatinine and cystatin C. Nephrol Dial Transplant. 2017;32(3):497–507. doi: 10.1093/ndt/gfw425 28089986 10.1093/ndt/gfw425 PMC5837496

[29] Kottgen A, Selvin E, Stevens LA, Levey AS, Van Lente F, Coresh J. Serum cystatin C in the United States: the Third National Health and Nutrition Examination Survey (NHANES III). Am J Kidney Dis. 2008;51(3):385–94. doi: 10.1053/j.ajkd.2007.11.019 18295054 10.1053/j.ajkd.2007.11.019

[30] Svensson-Farbom P, Ohlson Andersson M, Almgren P, Hedblad B, Engstrom G, Persson M, et al Cystatin C identifies cardiovascular risk better than creatinine-based estimates of glomerular filtration in middle-aged individuals without a history of cardiovascular disease. J Intern Med. 2014;275(5):506–21. doi: 10.1111/joim.12169 24279862 10.1111/joim.12169

[31] Glassock RJ, Rule AD. Optimally predicting mortality with kidney function markers is not the same as optimally determining how kidney function predicts mortality. Nephrol Dial Transplant. 2017;32(4):585–7. doi: 10.1093/ndt/gfx007 28339938 10.1093/ndt/gfx007 PMC6680095

[32] Grubb A, Lindstrom V, Jonsson M, Back SE, Ahlund T, Rippe B, et al Reduction in glomerular pore size is not restricted to pregnant women. Evidence for a new syndrome: ‘Shrunken pore syndrome’. Scand J Clin Lab Invest. 2015;75(4):333–40. doi: 10.3109/00365513.2015.1025427 25919022 10.3109/00365513.2015.1025427 PMC4487590

[33] Matsushita K, Coresh J, Sang Y, Chalmers J, Fox C, Guallar E, et al Estimated glomerular filtration rate and albuminuria for prediction of cardiovascular outcomes: a collaborative meta-analysis of individual participant data. Lancet Diabetes Endocrinol. 2015;3(7):514–25. doi: 10.1016/S2213-8587(15)00040-6 26028594 10.1016/S2213-8587(15)00040-6 PMC4594193

[34] Fox CS, Matsushita K, Woodward M, Bilo HJ, Chalmers J, Heerspink HJ, et al Associations of kidney disease measures with mortality and end-stage renal disease in individuals with and without diabetes: a meta-analysis. Lancet. 2012;380(9854):1662–73. doi: 10.1016/S0140-6736(12)61350-6 23013602 10.1016/S0140-6736(12)61350-6 PMC3771350

[35] James MT, Grams ME, Woodward M, Elley CR, Green JA, Wheeler DC, et al A Meta-analysis of the association of estimated GFR, albuminuria, diabetes mellitus, and hypertension with acute kidney injury. Am J Kidney Dis. 2015;66(4):602–12. doi: 10.1053/j.ajkd.2015.02.338 25975964 10.1053/j.ajkd.2015.02.338 PMC4594211

[36] Delanaye P, Glassock RJ, De Broe ME. Epidemiology of chronic kidney disease: think (at least) twice! Clin Kidney J. 2017;10(3):370–4. doi: 10.1093/ckj/sfw154 28617483 10.1093/ckj/sfw154 PMC5466090

[37] Benghanem Gharbi M, Elseviers M, Zamd M, Belghiti Alaoui A, Benahadi N, Trabelssi el H, et al Chronic kidney disease, hypertension, diabetes, and obesity in the adult population of Morocco: how to avoid “over”- and “under”-diagnosis of CKD. Kidney Int. 2016;89(6):1363–71. doi: 10.1016/j.kint.2016.02.019 27165829 10.1016/j.kint.2016.02.019

