# Supplementary material for: The clinical utility and cost impact of cystatin C measurement in the diagnosis and management of chronic kidney disease: A primary care cohort study
Source: PLoS Med. 2017 Oct 10;14(10):e1002400. doi: 10.1371/journal.pmed.1002400 (PMC5634538; doi:10.1371/journal.pmed.1002400)
Supplement: S1 Table — (DOCX) [file pmed.1002400.s003.docx]

**Supplementary Table 1**

| **Variable** | **Univariable**  **B (95% CI)** | **Adjusted for eGFR_creat_**  **B (95% CI)** | **Multivariable Model**  **B (95% CI)** |
| --- | --- | --- | --- |
| Male Sex | -4.58  (-6.10 to -3.05)* | 0.01  (-1.21 to 1.23) |  |
| Age | -6.30  (-6.99 to -5.60)* | -2.76  (-3.38 to -2.14)* | -3.21  (-3.84 to -2.58)* |
| Current Smoker | -2.45  (-6.02 to 1.13) | -4.56  (-7.32 to -1.81)* | -7.80  (-11.14 to -4.46)* |
| Diabetes | -5.88  (-7.88 to -3.89)* | -2.06  (-3.63 to -0.49)* | -1.56  (-4.89 to 1.77) |
| Haemoglobin | 3.26  (2.52 to 4.00)* | 0.98  (0.39 to 1.58)* | 0.65  (0.01 to 1.29)* |
| Corrected Calcium | 0.38  (-0.38 to 1.14) | -0.12  (-0.70 to 0.47) |  |
| Phosphate | -0.65  (-1.41 to 0.16) | -0.16  (-0.75 to 0.43) |  |
| Albumin | 3.06  (2.32 to 3.80)* | 1.85  (1.27 to 2.44)* | 1.05  (0.45 to 1.65)* |
| Bicarbonate | 2.27  (1.52 to 3.03)* | 0.55  (-0.05 to 1.15) |  |
| Total Cholesterol | 2.06  (1.31 to 2.81)* | 0.34  (-0.26 to 0.93) |  |
| Urate | -6.22  (-6.92 to -5.52)* | -1.90  (-2.56 to -1.25)* | -1.92  (-2.58 to -1.26)* |
| BMI | -1.66  (-2.41 to -0.90)* | -1.61  (-2.18 to -1.03)* | -1.50  (-2.11 to -0.90)* |
| Waist Hip Ratio | -3.31  (-4.05 to -2.57)* | -1.03  (-1.63 to -0.44)* | -0.20  (-0.82 to -0.42) |
| Previous CVD | -4.14  (-5.94 to -2.33)* | -1.04  (-2.45 to 0.38) |  |
| Ever Smoked | -3.39  (-4.90 to -1.88)* | -1.35  (-2.52 to -0.17)* |  |
| SBP | -0.63  (-1.39 to 0.12) | -0.12  (-0.71 to 0.46) |  |
| DBP | 3.31  (2.57 to 4.05)* | 1.27  (0.68 to 1.87)* | 0.57  (-0.04 to 1.17) |
| Log(uACR) | -3.76  (-4.49 to -3.03)* | -1.21  (-1.81 to -0.65)* | -0.81  (-1.41 to -0.22)* |
| Thyroid disorder | 0.33  (-1.95 to 2.61) | -1.02  (-2.78 to 0.75) |  |
| Log(hsCRP) | -3.22  (-3.96 to -2.49)* | -1.82  (-2.41 to -1.24)* | -0.73  (-1.34 to -0.22)* |
| All parameter estimates given per standard deviation for continuous variables  Multivariable Model 1 also adjusted for eGFR_creat_  Body Mass Index (BMI), Cardiovascular Disease (CVD), Diastolic Blood Pressure (DBP), Creatinine-based estimated glomerular filtration rate (eGFR_creat_), Cystatin C-based estimated glomerular filtration rate, High sensitivity C Reactive Protein, Systolic blood pressure (SBP), Urine Albumin to Creatinine ration (uACR) | | | |
