# Supplementary material for: The clinical utility and cost impact of cystatin C measurement in the diagnosis and management of chronic kidney disease: A primary care cohort study
Source: PLoS Med. 2017 Oct 10;14(10):e1002400. doi: 10.1371/journal.pmed.1002400 (PMC5634538; doi:10.1371/journal.pmed.1002400)
Supplement: S1 Text — (DOC) [file pmed.1002400.s005.doc]

**PROTOCOL: Clinical impact of adopting a new equation utilising cystatin C and creatinine to estimate glomerular filtration rate for diagnosis and risk prediction in older people with chronic kidney disease in primary care**.

## Aims and Objectives

*Background:* Worldwide adoption of a definition and classification system for CKD based on GFR [1] has focussed attention on the limitations of methods used to measure or estimate GFR. There is particular concern regarding potential harms to older people resulting from an incorrect diagnosis of CKD due to an inaccurate estimate of GFR [2]. These include unnecessary hospital visits and investigations, adverse effects of unnecessary medication, anxiety and financial implications (increased insurance costs etc). UK laboratories currently measure creatinine, a molecule produced by muscle cells and excreted by the kidneys, and use the MDRD equation to estimate GFR from serum creatinine [3]. Nevertheless, there are important non-GFR determinants of serum creatinine that may adversely affect this estimated GFR including muscle mass, diet and drug treatment. Moreover, the MDRD equation has been shown to underestimate true GFR at values above 60ml/min/1.73m^2^, the threshold for diagnosis of CKD [3]. These limitations are particularly relevant in older people because of age-related decline in muscle mass and because many older people with CKD have true GFR values only slightly below 60ml/min/1.73m^2^ [4,5]. A new equation, CKD-EPI [6], performs better than the MDRD and may replace MDRD in routine practice but remains limited by its reliance on creatinine [7]. Cystatin-C, a molecule produced by all nucleated cells, has been proposed as an alternative to creatinine [8] but GFR estimated from cystatin-C has not been shown to be more accurate than GFR estimated from creatinine, though cystatin C is a better predictor of death and cardiovascular events in older people [9]. Another study has reported that CKD confirmed by creatinine, cystatin-C and albuminuria is a better predictor of outcomes than if CKD is diagnosed using creatinine and albuminuria [10]. An equation has been developed that uses both serum creatinine and cystatin-C to estimate true GFR with improved precision and greater accuracy [11] but the impact of introducing cystatin C to confirm the diagnosis of CKD in routine practice is unknown.

*Aims:*

1. To compare estimates of glomerular filtration rate (GFR) derived from a new equation using cystatin-C and creatinine with the current standard equations (MDRD and CKD-EPI) that use only creatinine in a population of predominantly older people (mean age 73±9 years) previously diagnosed with CKD stage 3 recruited from Primary Care in the UK.
2. To evaluate the effect of use of the new creatinine-cystatin-C equation for estimating GFR on the frequency of diagnosis of CKD stage 3 and reclassification of people previously diagnosed with CKD in a cohort of predominantly older people recruited from Primary Care in the UK.
3. To compare the socio-demographic, lifestyle and clinical characteristics of those reclassified as not having CKD versus those with CKD confirmed by the combined equation.
4. To compare the performance of estimated GFR derived from the new creatinine-cystatin-C equation with the MDRD or CKD-EPI equations to predict important CKD-related risks (all-cause mortality, cardiovascular events, progression of CKD and acute kidney injury)
5. To explore the health economic impact of introducing cystatin-C measurement in combination with creatinine to estimate GFR in clinical practice.

**Study Design and Methodology:**

*Patients:*

The proposed study will be conducted in a large cohort (n=1741) of people aged >18 years with CKD stage 3 (GFR 30-59ml/min/1.73m^2^ on 2 occasions at least 3 months apart, prior to inclusion) recruited from 32 general practitioner (GP) surgeries as part of the Renal Risk in Derby (RRID) study from July 2008 to March 2010. The study population has been characterised in detail (mean age 73±9 years; 60% female; 98% Caucasian; 17% with diabetes; 88% with hypertension; mean MDRD estimated GFR 52.5±10.4ml/min/1.73m^2^; median (IQR) urine albumin to creatinine ratio 0.33 (0-1.5) mg/mmol)[5,12-15]. Importantly, the cohort is comprised predominantly of people with an estimated GFR (eGFR) in the range 45-59ml/min/1.73m^2^ (76%) and approximately half (47%) are aged 75 years or older [5]. The study is therefore well suited to evaluate the recommendation that people with a diagnosis of CKD based on a creatinine-based eGFR of 45-59ml/min/1.73m^2^ should have the diagnosis confirmed by a GFR estimated with the creatinine-cystatin-C equation [11].

At inclusion participants gave their written informed consent to participate in an observational study for up to 10 years. The proposed study procedures all fall within the scope of the consent previously given and participants will therefore not be required to provide additional consent. Participants underwent clinical assessment and serum as well as urine biochemistry were performed at baseline and after 1 year, with a follow-up rate of 96%. With the participants consent additional serum and urine samples were obtained and have been stored at -80 C for further biochemical assays as anticipated in the protocol. Per protocol assessments are due to be performed after 5 years, commencing July 2013 and will comprise:

*Questionnaires:* Participants will be asked to complete a self-administered questionnaire providing detailed medical history since their last study visit including hospital admissions and cardiovascular events as well as a list of current medication.

*Anthropomorphic and Clinical Assessment:* The following measurements will be performed: height, weight, body mass index, hip to waist ratio, blood pressure.

*Laboratory Assays:* GFR will be estimated from a standardised serum creatinine measurement using the modified 4-variable MDRD equation [16] and the CKD-EPI equation [6]. Subjects will be asked not to eat meat for 12 hours prior to the blood tests but will not be required to fast. Further blood tests will include: urea and electrolytes, calcium, phosphate, albumin, bicarbonate, uric acid, lipid profile, blood glucose, full blood count. Serum cystatin-C will be measured on stored serum specimens from the baseline and year-1 visits as well as specimens obtained at the year-5 visits using an immunoturbidometric assay that has been calibrated to the international reference material ERM-DA471/IFCC.63. Urine will be assessed by dipstick testing and analysis of three consecutive early morning specimens for total protein, albumin and creatinine. Routine blood and urine tests will be performed by the Clinical Chemistry Laboratory at Royal Derby Hospital. Cystatin-C assays will be performed as described above at the Department of Clinical Biochemistry, Oxford University Hospitals NHS Trust.

*Protocol:* Study visits will take place at GP Surgeries or at alternative venues including the Hospital Trust, if this is more convenient for participants. Year-5 results will be reviewed by a Consultant Nephrologist and GPs will be given written advice regarding management and the need for referral. This was previously done for results from the baseline and year-1 visits. Participants have been registered with the Medical Research Information Service (MRIS), a branch of the NHS Information service to obtain a record of all deaths including date and cause of death. Data regarding other cardiovascular and renal endpoints will be collected from patient self-reported questionnaires,

linkage to Hospital Episode Statistics data (HES) for details of any hospitalisations, .. Renal Unit databases in Derby, Nottingham and Chesterfield to confirm progression of CKD to the point of needing dialysis or renal transplantation, GP electronic records to confirm any details if required.

*Definition of study outcomes:*

1. All-cause mortality – death from any cause as reported by MRIS.
2. Cardiovascular event – myocardial infarction, stroke, transient ischaemic attack, any revascularisation procedure, amputation due to peripheral vascular disease, or aortic aneurysm.
3. Cardiovascular mortality – death due to any cardiovascular event as defined above in point 2 and recorded by MRIS as cause of death.
4. Progression of CKD over 5 years – patients with GFR decline in the top quintile for the study population[17].
5. Acute Kidney Injury (AKI) – AKI stages 1-3 as defined by the internationally accepted Acute Kidney Injury Network classification system [18].

Adjudication of cardiovascular and AKI end-points will be performed by a panel comprising three consultant nephrologists.

*Statistical Analysis:*

Descriptive statistics will be used to summarise data at each time point.

Aim 1. Estimated GFR values obtained with the new creatinine-cystatin-C equation will be compared to the MDRD eGFR using a paired t-test to compare the mean values and by Bland-Altman plots. The intraclass correlation coefficients (95% Confidence Interval) will be used to check the agreement of eGFR estimates by different equations. The analysis will be repeated in clinically important subgroups including participants under 75 years versus 75 years or older, males versus females and those with diabetes versus those without. The change in eGFR over 5 years determined by each of the GFR estimating equations will be similarly compared.

Aim 2. Patients will be classified at baseline as: not having CKD (GFR≥60ml/min/1.73m^2^ and no albuminuria), CKD stage 1-2 (GFR≥60ml/min/1.73m^2^ with albuminuria), CKD stage 3A (GFR 59-45ml/min/1.73m^2^), CKD stage 3B (GFR 44-30ml/min/1.73m^2^) or CKD stage 4 (GFR 29-15ml/min/1.73m^2^) based on eGFR derived from the MDRD and new creatinine-cystatin-C equation. The number and proportion of reclassifications will be derived (with 95% CI). This process will be repeated for the year 1 and year 5 data.

Aim 3. We will compare baseline socio-demographic, lifestyle and clinical characteristics of those reclassified as not having CKD versus those who remain as CKD and subsequent event rates.

Aim 4. We will compare all study outcomes (above) over 5 years in those reclassified as not having CKD by the combined equation versus those with CKD confirmed using Kaplan Meier plots. Cox proportional hazards models will be developed to compare hazard ratios for all-cause mortality, cardiovascular mortality, cardiovascular events and AKI using eGFR derived from the MDRD and combined equations.

Similarly, logistic regression analysis will be used to explore eGFR as an independent predictor of CKD progression over 5 years (top quintile of GFR decline versus rest). We will compare the performance of prognostic models using eGFR derived from the MDRD versus combined equation with respect to discrimination (C-statistic derived from ROC curves) and calibration (Hosmer Lemeshow test, net reclassification index (NRI)). We will repeat these analyses using change in GFR (estimated by MDRD or combined equations) and albuminuria over the first year of follow-up as independent variables as well as baseline values to test whether change over the first year adds to the prognostic model. Finally, we will investigate the prognostic value of categorising people according to GFR (<60 versus ≥60) and albuminuria as previously reported [10] and compare the effect of using GFR estimated by the combined versus MDRD equation.

Aim 5. A cost consequences approach (from an NHS perspective) will explore changes in resource use due to the different pattern of CKD diagnoses resulting from use of the new equation. This would include the potential reduction in routine monitoring visits and testing for those no longer classified as having CKD. It would be harder to define and cost changes in treatment due to the change in diagnoses in this cohort. The cost consequences approach will enable any savings due to changed diagnostic pattern to be compared with the additional laboratory costs of testing for cystatin-C. The resource use and outcomes data would inform a cost effectiveness model of CKD diagnosis, treatment and outcomes using different eGFR equations which we would intend to develop as a subsequent study.

As it is likely that the MDRD equation will be replaced in international guidelines and clinical practice by the CKD-EPI equation during the period of the proposed study, each of the above analyses will be repeated to compare the performance of creatinine-cystatin-C equation with the CKD-EPI equation.

*Sample Size/Statistical Power:*

The original cohort of 1741 patients recruited into the Risk in Derby (RRID) study from July 2008 to March 2010 will be included. It is expected that the mortality rate at 5 years will be 18.3%, based on a projection of the mortality rate to date (8.1% at April 2012) and assuming 60 new deaths per year. Based on 80% power and 5% significant level, the smallest significant Hazard Ratio (HR) that can be observed in the Cox Regression analysis will be 1.17 at 5 years follow up.

## Outcomes and Outputs

The results of this study will be important in informing a decision on whether or not to introduce cystatin-C together with creatinine as a measure of GFR to confirm a diagnosis of CKD in routine clinical practice. If our results confirm that the combined equation reduces the number of people diagnosed as having CKD, those reclassified as not having CKD will be spared anxiety and the need for further investigation or follow-up. They will also be spared the financial consequences of having a diagnosis of CKD including difficulties obtaining life or travel insurance. These issues are particularly important in older people, who have the highest prevalence of CKD [4] and are most frequently labelled as having CKD based on a small reduction in eGFR alone [5]. In addition the NHS will save the cost of further investigation and life long follow-up. Furthermore, payments made to GPs for achieving treatment targets under the QoF initiative are proportional to the number of patients on their CKD register. Thus reduced numbers diagnosed with CKD will result in reduced QoF payments. Given the large number of people diagnosed with CKD (1.81 million people with CKD stage 3-5 in England in 2009-2010 [19], there is potential for considerable savings nationally. Furthermore if improved risk prediction is confirmed, it will facilitate improved identification of people at high risk of CKD-related complications. This in turn will allow interventions including lower blood pressure targets and statin therapy to be directed at high-risk individuals while sparing low risk people unnecessary treatment. Accurate risk stratification is particularly important in older people because they are at highest absolute risk of CKD-related adverse outcomes [20] but also are increasingly exposed to greater medical intervention without adequate ascertainment of the risks versus benefits [21]. Unfortunately older people have frequently been excluded from studies of CKD, despite being the group most affected. Improved ability to select high-risk persons for inclusion in randomised controlled trials will also reduce the number of participants required and therefore reduce future research costs.

## Timetable and Milestones

April 2013 Dunhill Trust grant submission

March - June 2013 Staff recruitment; preparation; contracts

July 2013 Commence year-5 study visits

March 2015 Complete year-5 study visits

September 2015 Complete biochemical analyses

Sept – Dec 2015 Data verification, management and cleaning.

December 2015 Lock database

December 2015 - March 2016 Statistical analysis

June 2016 Final report; publications; dissemination of results

## Dissemination Plan

We plan to disseminate the results as widely as possible. A lay summary of results will be sent to participants who requested it (by post or by e-mail) and the results will be presented at a kidney patients’ forum by a team including the patient representative on the study management group. We will present a summary of the study in the Trust’s publications for members (“Interactions”) and General Practitioners (“GP Brief”) and offer these for publication in the local and national press. A detailed report will be sent to the National Kidney Federation (the largest kidney patient organisation in the UK), the participating GP surgeries, the East Midlands Renal Network and the National Clinical Director for Kidney Care. The findings will also be presented at national (British Renal Society and Renal Association) and international (European Renal Association and American Society of Nephrology) scientific conferences. In addition we plan to publish our findings in a high-impact international peer-reviewed journal.

## Justification of Costs

1. A research fellow will perform the year-5 study visits as well as data entry and verification, obtain data from HES and MRIS and perform data analysis. Based on our experience during the recruitment and year-1 phases of this study, these tasks could not reasonably be performed by conventional research nurse. We have therefore recruited Dr Adam Shardlow, Specialist Registrar in Nephrology, to fulfil this role for the duration of the year-5 follow-up programme.
2. An administrative assistant/project manager will liase with GP practices to schedule visit times, send out appointment letters, collate results and produce feedback letters to GPs. Based on the large number of participants and the experience of conducting the baseline and year-1 visits, we are confident that this is the amount of administrative time required.
3. A statistician will be required to supervise management of the large database and the complex analyses required.
4. The expertise of a health economist will be essential to explore the health economic implications of the data and inform a recommendation on whether or not to introduce cystatin-C assays and the combined equation for estimating GFR into routine practice.
5. Funding is required to pay for the cystatin-C assays on serum stored at baseline and year-1 visits as well as year-5 specimens. Routine laboratory assays will be paid for by the NHS.

This project represents extremely good value for money because the considerable costs of establishing the cohort and conducting the year-1 follow-up visits as well as investigations have already been met. The fact that the cohort was established in 2008-2010, means that the study will benefit from outcomes data over a far longer period than would have been achieved during the proposed three year period of funding than if the cohort was established during the funding period.

## Roles of Members of the Research Team

**Maarten Taal** is principal investigator for the Renal Risk in Derby study. He will continue to lead the study, supervise the research fellow and be responsible for assessing participant data as well as providing individualised feedback to GPs.

**Christopher McIntyre** is Professor of Nephrology with extensive experience in clinical research projects focussing on the cardiovascular complications of CKD. He was closely involved in protocol development and will continue to provide advice as well as assisting with analysis and interpretation of results.

**Richard Fluck** is a consultant nephrologist and National Clinical Director for Renal Medicine. He contributed to protocol development and will continue to provide advice as well as assist with interpretation of results, particularly within a national context.

**Paul Roderick** is Professor of Public Health with extensive experience in the clinical epidemiology of CKD. He served on the Renal Advisory Group at the Department of Health and is on the advisory group updating the NICE guidance on the management of CKD. He contributed substantially to protocol development and will continue to provide advice as well as assisting with analysis and interpretation of results.

**David Young** is a general practitioner who contributed to the establishment of the RRID study. He provided an essential Primary Care perspective to assist the design of the study and he will continue to serve on the study management group. His contribution will ensure that the study addresses issues that are important to GPs and that data are presented in a manner that is relevant to GPs.

**Apostolos Fakis** is an experienced statistician in planning and analysing funded trials and in peer reviewing for NIHR programs. He has assisted with the statistical aspects of the RRID study design and analysis. He will lead the management of the large database and the statistical analysis.

**James Raftery** is a Professor of Health Technology Appraisal at the University of Southampton and chair of the NIHR Evaluation Trials and Studies Coordinating Centre (NETSCC). He will lead the health economic analysis.

**Ian Hazel** is a patient with personal experience of having CKD. His involvement in the study management group will ensure that the research questions explored are relevant to patients’ concerns and needs and that data are presented in a manner that is accessible to patients.

## Research Environment

The Derby Renal Research Group is based within the Division of Graduate Entry Medicine and Medical Science (HoD Prof McIntyre) part of the School of Medicine (University of Nottingham). The group consists of Professor McIntyre, two Consultant Nephrologists (Hon. Assoc Prof- Dr MW Taal, Dr NM Selby) with research dedicated time and 11 research fellows, supported by nursing, technical and administrative staff. This Renal Research Group runs a highly successful integrated research program specifically directed at the physiologically frail patient with kidney disease (covering CKD, dialysis and AKI). Studies include basic science, clinical science and direct translation to developing novel therapies to reduce the high rate of morbidity and mortality in these patient groups. We have published more than 100 peer-reviewed articles in the last five years alone. Since 2006 our group has raised significant amounts of research-dedicated income (total around £2m) from external competitive sources (including NIHR, KRUK, Heart UK and BHF). Our research group currently provides the PI on 11 UKCRN portfolio registered studies. Professor McIntyre is responsible for principle supervision of 28 higher degree PhD/DM students in the last 8 years, with 100% successful matriculation. Derby Hospitals NHS Foundation Trust already acts as the sponsor for the RRID Study and the Research and Development Department has a robust research governance system in place to ensure that the study is conducted according to the principles of “Good Clinical Practice” and the Declaration of Helsinki. As part of these arrangements the study was independently audited by QED Clinical Services in November 2009.

**References:**

1. Kidney Disease Outcomes Quality Initiative. K/DOQI clinical practice guidelines for chronic kidney disease: evaluation, classification, and stratification. Am J Kidney Dis 2002; 39:S1-266.

2. Winearls CG, Glassock RJ. Dissecting and refining the staging of chronic kidney disease. Kidney Int 2009; 75:1009-1014.

3. Levey AS, Bosch JP, Lewis JB, Greene T, Rogers N, Roth D. A more accurate method to estimate glomerular filtration rate from serum creatinine: a new prediction equation. Modification of Diet in Renal Disease Study Group. Ann Intern Med 1999; 130:461-470.

4. **Roderick P**, Roth M, Mindell J. Prevalence of chronic kidney disease in England: Findings from the 2009 Health Survey for England. J Epidemiol Comm Health 2011; 65:A12.

5. McIntyre NJ, Fluck RJ, **McIntyre CW, Taal MW**. Risk profile in chronic kidney disease stage 3: older versus younger patients. Nephron Clin Pract 2011; 119:c269-276.

6. Levey AS, Stevens LA, Schmid CH, Zhang YL, Castro AF, 3rd, Feldman HI, Kusek JW, Eggers P, Van Lente F, Greene T, Coresh J. A new equation to estimate glomerular filtration rate. Ann Intern Med 2009; 150:604-612.

7. Matsushita K, Mahmoodi BK, Woodward M, Emberson JR, Jafar TH, Jee SH, Polkinghorne KR, Shankar A, Smith DH, Tonelli M, Warnock DG, Wen CP, Coresh J, Gansevoort RT, Hemmelgarn BR, Levey AS. Comparison of risk prediction using the CKD-EPI equation and the MDRD study equation for estimated glomerular filtration rate. JAMA 2012; 307:1941-1951.

8. Inker LA, Okparavero A. Cystatin C as a marker of glomerular filtration rate: prospects and limitations. Curr Opin Nephrol Hypertens 2011; 20:631-639.

9. Shlipak MG, Sarnak MJ, Katz R, Fried LF, Seliger SL, Newman AB, Siscovick DS, Stehman-Breen C. Cystatin C and the risk of death and cardiovascular events among elderly persons. N Engl J Med 2005; 352:2049-2060.

10. Peralta CA, Shlipak MG, Judd S, Cushman M, McClellan W, Zakai NA, Safford MM, Zhang X, Muntner P, Warnock D. Detection of chronic kidney disease with creatinine, cystatin C, and urine albumin-to-creatinine ratio and association with progression to end-stage renal disease and mortality. JAMA 2011; 305:1545-1552.

11. Inker LA, Schmid CH, Tighiouart H, Eckfeldt JH, Feldman HI, Greene T, Kusek JW, Manzi J, Van Lente F, Zhang YL, Coresh J, Levey AS. Estimating glomerular filtration rate from serum creatinine and cystatin C. N Engl J Med 2012; 367:20-29.

12. McIntyre NJ, Fluck RJ, **McIntyre CW, Taal MW**. Skin autofluorescence and the association with renal and cardiovascular risk factors in chronic kidney disease stage 3. Clin J Am Soc Nephrol 2011; 6:2356-2363.

13. Evans PD, McIntyre NJ, Fluck RJ, **McIntyre CW, Taal MW**. Anthropomorphic measurements that include central fat distribution are more closely related with key risk factors than BMI in CKD stage 3. PLoS One 2012; 7:e34699.

14. Fraser SD, **Roderick PJ**, McIntyre NJ, Harris S, **McIntyre CW**, Fluck RJ, **Taal MW**. Socio-economic disparities in the distribution of cardiovascular risk in chronic kidney disease stage 3. Nephron Clin Pract 2013; 122:58-65.

15. McIntyre NJ, Fluck RJ, **McIntyre CW**, Fakis A, **Taal MW**. Determinants of arterial stiffness in chronic kidney disease stage 3. PLoS One 2013; 8:e55444.

16. Levey AS, Coresh J, Greene T, Stevens LA, Zhang YL, Hendriksen S, Kusek JW, Van Lente F. Using standardized serum creatinine values in the modification of diet in renal disease study equation for estimating glomerular filtration rate. Ann Intern Med 2006; 145:247-254.

17. Halbesma N, Jansen DF, Heymans MW, Stolk RP, de Jong PE, Gansevoort RT. Development and validation of a general population renal risk score. Clin J Am Soc Nephrol 2011; 6:1731-1738.

18. Mehta RL, Kellum JA, Shah SV, Molitoris BA, Ronco C, Warnock DG, Levin A. Acute Kidney Injury Network: report of an initiative to improve outcomes in acute kidney injury. Crit Care 2007; 11:R31.

19. Kerr M, Bray B, Medcalf J, O'Donoghue DJ, Matthews B. Estimating the financial cost of chronic kidney disease to the NHS in England. Nephrol Dial Transplant 2012; 2012:5.

20. Hallan SI, Matsushita K, Sang Y, Mahmoodi BK, Black C, Ishani A, Kleefstra N, Naimark D, **Roderick P**, Tonelli M, Wetzels JF, Astor BC, Gansevoort RT, Levin A, Wen CP, Coresh J. Age and association of kidney measures with mortality and end-stage renal disease. JAMA 2012; 308:2349-2360.

21. **Taal MW**. Screening for chronic kidney disease: preventing harm or harming the healthy? PLoS Med 2012; 9:e1001345.
